# The Treatment of Phthisis by Antiseptic Inhalations

**Published:** 1885-11

**Authors:** Rene Couetoux, F. R. Campbell


### Translations.

### The Treatment of Phthisis by Antiseptic Inhalation.

Dr. Rene Couetoux.

Translated from *Bulletin Gen. de Therapeutique*, by F. R. Campbell, M. D.

The diseases of the respiratory organs are especially susceptible to medicines introduced into the system with the inspired air. This method of medication presents, indeed, the most direct means of reaching the different parts of the respiratory system. Phthisis is considered contagious by a great number of physicians, and, on this account, presents to the therapeutist the two great indications for antisepsis to which I have called your attention: (1) the treatment of the patient himself; (2) the modification by antiseptics of the atmosphere which surrounds the patient. I will be as brief as possible in discussing this subject, because one cannot, in a few months, appreciate the exact clinical value of theoretical notions respecting a disease so prolonged, and subject to such frequent changes, as phthisis.

I commenced the treatment of my patients affected with phthisis January 21, 1885, and I will now sum up the results obtained before the 20th of May, a period of only four months. Up to April 21st, my treatment appeared to be surprisingly efficacious. All my patients improved; the hectic fever, with all the associated symptoms of night sweats, anorexia, diarrhœa, and loss of flesh, seemed to disappear. All improved in spirits and in weight, and the pulmonary lesions themselves diminished in severity and in extent. I have especially observed a young woman, phthisical since her fourteenth year, having had, during this period, a miscarriage and two confinements, who, after the birth of her last child, was affected with hectic fever, and death now seemed to be imminent. Well! On the 12th of April, she did not cough, except when, during the day, she engaged in some very tiresome labor, or, at night, she uncovered herself to care for her infant girl. Her expectorations had become almost entirely mucous and not very abundant, although they had previously been copious and purulent. She arose about seven or eight o'clock in the morning and retired about nine or ten at night. She attended alone to her household duties, and had assumed an appearance of better health than for some years previous. The pulmonary lesions, although very grave and extensive at first, became more and more limited, and, consequently, strong hopes of her recovery were entertained.

But, about this same date, (April 12th,) there was a period of cold and rainy weather, during which all my patients were obliged to keep their rooms, and their disease assumed a grave condition with a rapidity and uniformity quite remarkable. The pulmonary lesions began to extend, the cough became fatiguing, and the expectorations abundant. The strength, regained for an instant, disappeared with great rapidity; but the hectic fever did not, as a rule, assume its former intensity, and the night sweats remained moderate.

To-day, the young woman keeps her bed almost continually; she becomes more and more despondent, and tells her friends that she will soon die. But the fever is not so intense and the night sweats are moderate. Another of my patients often became unmanageable, not being able to understand how a method of treatment that she had never seen before could be effective or rational. I observed with interest that her hectic fever ceased or came again, as she resumed or stopped the medicated inhalations.

As far as prophylaxsis is concerned, I would willingly compare the results which this method of treatment is able to accomplish for a case of phthisis, with those I have obtained with the same kind of medication in an epidemic of diphtheria.

I can already support my theory with a partial demonstration: The husband of the young woman previously mentioned was affected with alarming symptoms at the time I began to treat his wife by inhalations. This man, who is very poor in this world's goods, was not slow in recovering his usual health; he is not troubled by the cough, oppression and expectoration. When affected recently with a slight bronchitis, he recognized, himself, the difference between this accident and the process of continued decline, of which he formerly thought himself the victim. At the same time, the health of the two daughters has been improved. They have partly lost their strumous appearance, and I have seen in the elder a conjunctivitis disappear and return several times, as the inhalations were resumed or stopped. I *suppose*, then (and I use the word suppose designedly)—I suppose that the health of this man and that of his children has been improved because the air in which they live has been disinfected. I give below a resumé of all my observations, excepting for a small number of mild cases of a more recent date, and cases of chronic bronchitis with emphysema, who have not been cured but are generally improved. If I have not cured my patients, almost all have been greatly benefited. A few days ago, I saw a young girl, sixteen years old, for whom a sympathizing lady friend, knowing in part my method of treating those affected with diseases of the chest, advised inhalations of turpentine. The poor child was badly affected; both lungs were diseased, an auscultation of the right lung showed a well-marked amphoric souffle. In spite of this wretched condition of affairs, the patient enjoyed these inhalations, which had quieted her cough, diminished her expectorations, and increased her strength. She had just stopped this exclusive treatment, which had finally produced an excessive dryness of the bronchi. I advised her to resume the inhalations, but to change the substances used for medication.

In order to give an accurate idea of the clinical results which I have obtained, I will review the principal symptoms of chronic pulmonary affections, and state, concerning each, the modification produced by this treatment:

1. *Cough.* Every one knows that jockeys treat the cough of horses with fumigations of tar, and obtain excellent results. This treatment is too reasonable to be denied, and it is self-evident that, if irritating substances in the respiratory organs provoke cough, quieting substances will relieve it. I have just treated a child, five months old, for a slight bronchitis. Inhalations of tar were used, and an immediate improvement has been followed by a complete cure. The result obtained is very satisfactory, because I would not have dared administer tar by the stomach to a child so young. This case shows, also, the benefits which can be derived from fumigations and inhalations in the treatment of infantile diseases.

2. *Expectoration.* If we continued for a long time the use of certain substances by inhalations, especially the oil of turpentine, we obtain a diminution of the expectoration and a dryness of the bronchi, which becomes painful to the patient. This dryness could not be produced by the same medicines taken by the stomach, because this marked effect would be preceded by the feeling of nausea, and would then be prevented. For example, on the 27th of February, I heard râles in the left supraspinous fossa of one of my patients. It appeared as if the air penetrated into large cells whose walls, almost dry and covered over by a thick viscous liquid, separated with great difficulty. It is in such cases especially that inhalations with aromatic and emollient drugs are indicated, and procure for the patients a great and very rapid improvement.

The character of the expectoration is also modified. Under the influence of inhalations of various substances, such as the oil of turpentine, tar, carbolized glycerine and eucalyptus, they become tasteless and inodorous, though they were previously fetid. In the course of a few days, under the influence of carbolized glycerine and eucalyptus leaves, I have seen the sputa lose their purulent character and become entirely mucous. By this means, too, the sputa are disinfected at the place of their formation. It would be an interesting study to ascertain whether a microscopical examination would reveal the presence of the bacillus tuberculosis in as great number as before in sputa thus modified.

This powerful action of certain inhalations upon the quantity and quality of the expectoration, explains how cases of chronic catarrh with emphysema, and even bronchiectasis, are susceptible to this treatment. Inhalations of the fumes of eucalyptus leaves are very efficacious in these cases.

3. *Diarrhœa.* As this symptom is but little influenced by inhalations, I have often been asked if some remedy should not be given for the diarrhœa. I have observed that an aggravation of the pulmonary symptoms sometimes coincides with the sudden disappearance of the diarrhœal discharge. It is often necessary, however, to lessen this source of exhaustion; and I take this opportunity to mention a popular remedy, which I have heard lauded by many intelligent persons who deserve our confidence, and from which I have myself derived good effects in the treatment of my patients. I refer to confection of the fruit of the service berry, *Amelanchier Canadensis*, of which a tablespoonful a day is generally sufficient to stop a severe diarrhœa. One of my patients found it too irritating. I advised her to mix it with a little warm water and an equal quantity of confection of quince, and the result was thus very satisfactory.

From all that I have just said concerning the treatment of phthisis by continued inhalations, I conclude, simply, that this method presents important advantages and deserves the attention of the profession. It respects the digestive functions of exhausted patients, and when digestion can no longer be accomplished, the stomach refusing almost all nourishment and not tolerating any medicine, it permits us to take advantage of this valuable auxiliary, and procures for the patient the benefits of a rational and, at the same time, a very active treatment. It presents no obstacle to the employment, at the same time, of internal and revulsive medication. Finally, being within the reach of all classes of society, it can serve to assuage the cruel pains and diminish the prolonged sufferings of the poor. Mullen, hyssop, and branches of the spruce tree are three remedies which cost but little and are sufficient to meet the principal indications.

